# Correlative cryo-imaging of the cellular universe with soft X-rays and laser light used to track F-actin structures in mammalian cells

**DOI:** 10.1107/S2059798321010329

**Published:** 2021-11-29

**Authors:** Mohamed Koronfel, Ilias Kounatidis, Dennis M. Mwangangi, Nina Vyas, Chidinma Okolo, Archana Jadhav, Tom Fish, Phatcharin Chotchuang, Albert Schulte, Robert C. Robinson, Maria Harkiolaki

**Affiliations:** a Beamline B24, Diamond Light Source, Harwell Science and Innovation Campus, Didcot OX11 0DE, United Kingdom; b Institute of Molecular and Cell Biology, A*STAR (Agency for Science, Technology and Research), Biopolis, Singapore 138673, Singapore; c Research Institute for Interdisciplinary Science (RIIS), Okayama University, Okayama 700-8530, Japan; d School of Biomolecular Science and Engineering (BSE), Vidyasirimedhi Institute of Science and Technology (VISTEC), Rayong 21210, Thailand

**Keywords:** actin filaments, soft X-ray tomography, super-resolution microscopy, correlative imaging, structured illumination

## Abstract

Imaging of actin filaments is crucial due to the integral role that they play in many cellular functions such as intracellular transport, membrane remodelling and cell motility. Visualizing actin filaments has so far relied on fluorescence microscopy and electron microscopy/tomography. The former lacks the capacity to capture the overall local ultrastructure, while the latter requires rigorous sample preparation that can lead to potential artefacts, and only delivers relatively small volumes of imaging data at the thinnest areas of a cell. In this work, a correlative approach utilizing *in situ* super-resolution fluorescence imaging and cryo X-ray tomography was used to image bundles of actin filaments deep inside cells under near-native conditions. In this case, fluorescence 3D imaging localized the actin bundles within the intracellular space, while X-ray tomograms of the same areas provided detailed views of the local ultrastructure. Using this new approach, actin trails connecting vesicles in the perinuclear area and hotspots of actin presence within and around multivesicular bodies were observed. The characteristic prevalence of filamentous actin in cytoplasmic extensions was also documented.

## Abstract 2

A 3D cellular imaging platform developed at beamline B24 at Diamond Light Source has been used to identify cellular features such as filamentous actin in mammalian cells. This approach enabled virtual same-sample imaging of structures captured on separate microscopes through rigid transformation of 3D data *in silico*, bypassing the need for additional sample processing and ensuring artefact-free data correlation.

## 1. Introduction

Actin is one of the most abundant proteins in eukaryotic cells (Wu & Pollard, 2015 ▸). Filamentous actin (F-actin) monomers polymerize to form actin filaments, which are thin and flexible fibres that comprise a major component of the cytoskeleton of cells. Networks of actin filaments act as mechanical supports that control cell shape and movement, thus facilitating cell migration, division and phagocytosis. Furthermore, actin plays an integral role in other cellular functions, including intracellular transport (Calvo & Izquierdo, 2021 ▸; Villanueva *et al.*, 2016 ▸), vesicle budding (Traikov *et al.*, 2014 ▸; Picco *et al.*, 2018 ▸), cell–cell contact regulation (Dominguez & Holmes, 2011 ▸) and gene regulation (Louvet & Percipalle, 2008 ▸; Bunnell *et al.*, 2011 ▸), as well as other nuclear functions (Grosse & Vartiainen, 2013 ▸; Plessner & Grosse, 2015 ▸).

To fully understand the role of actin in these functions, it is crucial to visualize filamentous networks in the relevant volume of the cell under native conditions and unambiguously map their location with respect to local substructures, without inducing actin (de)polymerization due to sample handling. Actin filaments are the narrowest component of the cyto­skeleton, with an average diameter of 7 nm (Merino *et al.*, 2020 ▸), therefore their visualization within cells largely depends on the use of fluorescence microscopy techniques as well as electron microscopy and tomography (EM/ET) (Abdellatif *et al.*, 2019 ▸; Zhang *et al.*, 2020 ▸; Taylor *et al.*, 2019 ▸; Joosten *et al.*, 2018 ▸). However, fluorescence-based imaging probes can conceivably influence actin dynamics and therefore careful consideration should be paid to the amount or expression level of a given detection probe (Melak *et al.*, 2017 ▸). Moreover, as fluorescence imaging detects discrete signals from tagged intracellular targets, the information content achieved is limited to these targets only (as opposed to full capture of the ultrastructure that surrounds them). At the other extreme, electron microscopy, which provides excellent contrast for visualizing local ultrastructure at superior spatial resolution (3.1 Å resolution has been achieved with cryo-EM; Chou & Pollard, 2019 ▸), presents challenges in sample preparation (chemical fixation and sectioning are often required), while electron tomography is limited to the thinnest areas in a cell and therefore cannot shed light on cellular substructure close to and within the nucleus.

The above limitations of EM approaches can be overcome through the use of cryo soft X-ray tomography (SXT), which allows the imaging of whole cells without the need for sample processing or sectioning (Harkiolaki *et al.*, 2018 ▸; Kounatidis *et al.*, 2020 ▸; Groen *et al.*, 2021 ▸) as imaging takes place in the ‘water’ window (the spectral region between the C and O *K* edges). In this energy range SXT is not dependent on extraneous contrasting agents, but rather uses the differential absorption of X-ray light by carbon-rich cellular structures in order to generate contrast and image relatively large swathes of intracellular space (10^2^–16^2^ µm^2^) to spatial resolutions of 25–40 nm. At this resolution SXT faces a potential challenge in imaging actin, which is present either at low local concentrations (resulting in marginal carbon absorption above the background) or organized in thin radiating structures (with a signal spread over large areas, which reduces the overall signal-to-noise ratio). Therefore, a combinatorial approach employing SXT and high-resolution fluorescence imaging is required to fully capture F-actin organization in cells. In particular, 3D super-resolution fluorescence imaging is the most suitable method for such a correlative scheme as it allows imaging at resolutions beyond the diffraction limit of visible light, thus bridging the resolution gap between SXT and traditional fluorescence microscopy. Structured illumination microscopy (SIM) offers just such an advantage, with the added benefits of low dose requirements, limited sample handling and an available user-friendly implementation (Phillips *et al.*, 2020 ▸).

Here, we demonstrate the correlative use of SXT and SIM at cryogenic temperatures to visualize bundles of actin filaments within vitrified adherent mammalian cells. The imaging platform used was designed and commissioned at the correlative cryo-imaging beamline B24 at the UK synchrotron Diamond Light Source (Kounatidis *et al.*, 2020 ▸). This combination of methods allowed the visualization of F-actin using the fluorescence capabilities of the beamline, while the surrounding cellular structure was obtained through on-site X-ray imaging of the same area within the same samples. This approach resulted in the high-resolution native 3D imaging of actin bundles in large volumes within the cytoplasmic environment and allowed us to pinpoint hotspots and trails that highlight key events in the cytoplasm of mammalian cells.

## 2. Materials and methods

### 2.1. Sample preparation

Human osteosarcoma U2OS cells expressing LifeAct-mRFPruby (Riedl *et al.*, 2008 ▸) were a kind gift from E. Manser and Y. Baskaran (A*STAR, Singapore). Cells were grown adherent at 37°C in 5% fetal bovine serum (FBS) in complete Dulbeccos’ modified Eagle’s medium (DMEM) supplemented with penicillin/streptomycin. Cells were lifted from the plates using trypsin/ethylenediaminetetraacetic acid (EDTA) and were used to seed 3 mm carbon-coated EM grids (Quantifoil) in the same medium. Once they had adhered, they were allowed to grow to 60–70% confluence, whereupon the grids were removed from the medium, fiducialized using 250 nm diameter gold nanoparticles (BBI Solutions), gently blotted and vitrified by plunge-freezing into liquid nitrogen-cooled liquid ethane using an EM GP Automatic Plunge Freezer (Leica). The cryopreservation process immobilizes the sample in native conditions and provides resistance to radiation damage during imaging. Samples were thereafter stored in liquid nitrogen until retrieved for imaging. All samples were prepared following established protocols as documented previously (Okolo *et al.*, 2021 ▸).

### 2.2. Data collection and processing

Vitrified cells on grids were first transferred to the cryostage coupled to the cryoSIM microscope at beamline B24 for SIM data collection. Grids were initially mapped using the brightfield functionality of the setup, and brightfield *z* stacks and SIM stacks of areas of interest were then collected. The cryoSIM is fitted with a long working-distance air objective lens (100×, 0.9 NA, 2 mm working distance; Nikon) and delivers imaging at resolutions beyond the diffraction limit (∼200 nm). The data were collected using an excitation beam wavelength of 561 nm and an emission wavelength of 605 nm. Data were reconstructed with *SoftWoRx* 6.5.2 (GE Healthcare) using real optical transfer functions generated from 3D-SIM images of 175 nm single-colour fluorescent PS-Speck beads (Thermo Fisher Scientific) to produce super-resolution image stacks. The pixel sizes of the reconstructed data are 62.5 × 62.5 × 125 nm (*x* × *y* × *z*).

The same grids were then loaded onto an UltraXRM-S/L220c X-ray microscope (Carl Zeiss) at beamline B24 and first mapped using an inline visible-light objective. This microscope is maintained under a high vacuum of 10^−6^–10^−8^ mbar (due to the poor penetration depth of soft X-rays at atmospheric pressure) and at cryogenic temperature. The microscope consists of a capillary condenser, a zone-plate objective and a 1024B Pixis CCD camera (Princeton Instruments). Two zone plates were used in this study with an outermost zone width of 25 and 40 nm (which defines the resolution of the zone plates). The field of view was 10 × 10 and 16 × 16 µm for the 25 and 40 nm zone plates, respectively. For each sample, X-ray mosaics of the areas of interest were captured, followed by sample alignment and acquisition of tilt series. Series of X-ray images were collected using an incident beam of 500 eV at tilt angles from −55° to +70° in 0.2° increments. The total possible tilt range is −70° to +70° due to the microscope geometry, but for particular regions on the edge of the grids, such as that used here in this study, higher tilts on one side may not be possible as the sample becomes obstructed by the grid holder. The increment of 0.2° is the smallest mechanically possible increment and hence provides the maximum number of projections for the reconstructions. Higher increments are generally considered for samples that are more prone to radiation damage. Tilt series were aligned and 3D tomograms were then reconstructed using iterative reconstruction and/or weighted back-projection protocols in *IMOD* (Kremer *et al.*, 1996 ▸).

The microscopy platform used here has been fully documented previously (Kounatidis *et al.*, 2020 ▸; Phillips *et al.*, 2020 ▸; Ludin *et al.*, 2021 ▸; Vyas *et al.*, 2021 ▸; Groen *et al.*, 2021 ▸).

### 2.3. Data correlation

In order to match and overlay corresponding SIM and SXT imaging volumes, data need to be transformed in both 2D space (*x* and *y* directions) and 3D space (*z* direction). Sample grids are sequentially loaded from one microscope to the next, and as a result their orientation during data collection differs between SIM and SXT. Moreover, each method results in data of varying resolution (25–40 nm for SXT; 200 nm for SIM), pixel size (10 or 16 nm for SXT; 62.5 nm in *x* and *y* and 125 nm in *z* for SIM) and field of view (10^2^ or 16^2^ µm^2^ for SXT; 64^2^ µm^2^ for SIM). In order to fully align these data in 3D all of these parameters need to be accounted for, and for this we used the multidimensional registration software *eC-CLEM* (Paul-Gilloteaux *et al.*, 2017 ▸), which has been adapted for the purposes of our platform (Vyas *et al.*, 2021 ▸). The process involves the derivation of three translocation matrices accounting for any *x* and *y* shifts with a final manual alignment along the *z* axis. All data transformations are scalar/positional and no data deformation is allowed in order to ensure that the data remain faithful to the sample at the point of collection.

## 3. Results

### 3.1. Methodology development

**Figure 1:**
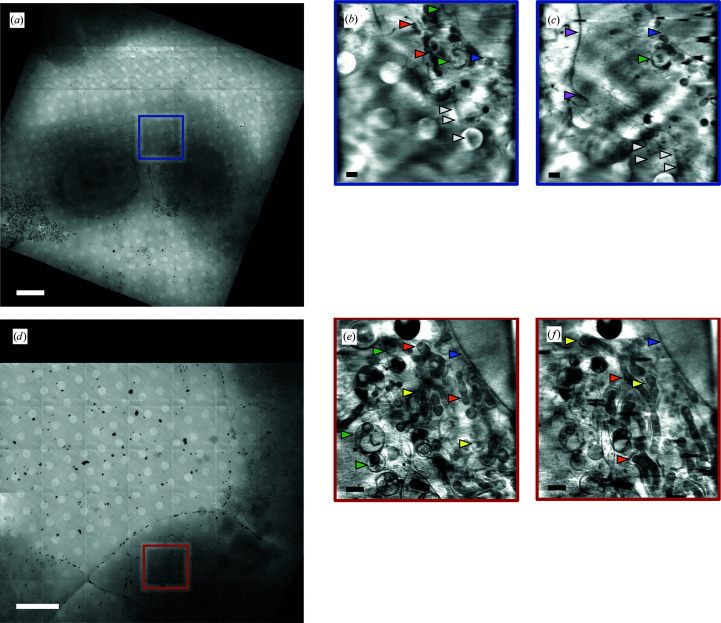
X-ray mosaics (7 × 7 frames) from areas of interest collected from two different grids bearing vitrified U2OS cells with (*a*) the 40 nm and (*d*) the 25 nm zone plate. Data were collected in the highlighted areas in (*a*) and (*d*) and representative slices from the reconstructed tomograms at different *z* heights can be seen in (*b*), (*c*), (*e*) and (*f*) (colour-coded to match the originating mosaic). The arrows used to highlight the cellular features are nuclear membrane in blue, outer cell membrane in purple, endoplasmic reticulum in yellow, mitochondria in orange, multivesicular bodies in green and actin bundles in white. Scale bars are 10 µm for (*a*) and (*d*) and 1 µm for (*b*), (*c*), (*e*) and (*f*).

X-ray tomography data were collected from adherent U2OS cells using either a 25 or a 40 nm zone plate as the objective lens for the transmission X-ray microscope at beamline B24 (Fig. 1 ▸). These produce different fields of view (10^2^ versus 16^2^ µm^2^) as well as different depths of focus (0.7 versus 1.9 µm), providing an exchange of image resolution (which was higher with the 25 nm zone plate) for a reduction in the overall area size that can be seen (which was larger with the 40 nm zone plate). In both of these modes the visualization of F-actin was challenging as elongated shadows traversing the cytoplasmic area (particularly clear in the 40 nm data) were presumed to represent X-ray absorption brought about by structured F-actin bundles but lacked any clear definition (Fig. 1 ▸). In data collected from crowded cytoplasmic regions heavily occupied by organelles and other membranous structures any association of features with the presence of F-actin was not possible even when the data were collected at the highest possible image resolution (25 nm). Hence, although a great amount of detail was evident in the acquired data, the low signal-to-noise profile of F-actin structures rendered their unambiguous localization impossible at times within the X-ray data.

**Figure 2:**
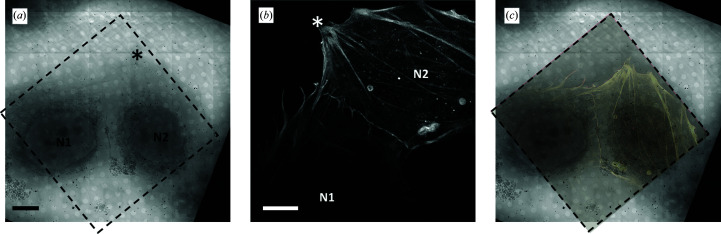
2D X-ray mosaic (*a*) from an area of interest and (*b*) the corresponding SIM data (maximum intensity projection along the *z* axis) showing the different features imaged by the two techniques: (*a*) carbon absorption from structured cellular features and (*b*) fluorescence signal from the filamentous actin components of the cytoskeleton. Each technique offers unique information that is otherwise inaccessible by its individual partner. The data appear as collected and have not been repositioned *in silico* to overlap. (*c*) Overlay of (*a*) and repositioned (*b*) to extract correlated content. The two nuclei of the cells present are denoted N1 and N2 and a star is used to point out the equivalent areas in the sample across the two modalities in order to aid the correct orientation. Scale bars are 10 µm.

To address this difficulty, super-resolution fluorescence imaging is the natural partner to provide further and fundamentally unique localization information. The SIM data collected only captured structural information as this pertained exclusively to filamentous actin (LifeAct technology uses a peptide that preferentially marks filamentous actin). Therefore, it is an excellent reporter of the native network of F-actin, although lacking information on the cellular ultrastructure that surrounds and contains it (Fig. 2 ▸).

**Figure 3:**
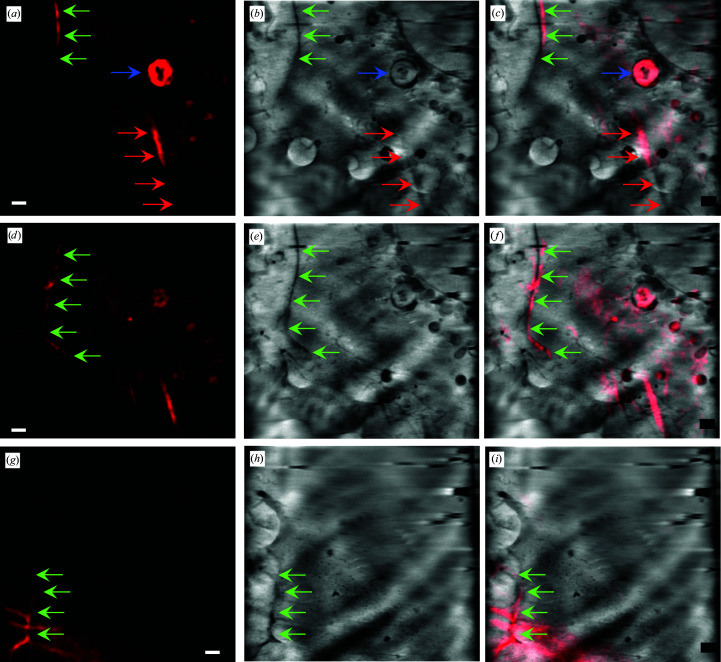
2D slices of imaging data along *z* starting from near the support surface (*a*, *b*), followed by slices from the mid-region of the sample (*d*, *e*) and finally the upper area of the sample (*g*, *h*). SIM data are shown in (*a*), (*d*) and (*g*) and the corresponding SXT data are shown in (*b*), (*e*) and (*h*). The correlated images are shown in (*c*), (*f*) and (*i*). All SXT data sets shown here were collected using the 40 nm zone plate. Red arrows indicate bundles of F-actin that are barely delineated in the SXT data but can be clearly identified based on the correlation of the associated fluorescence signal. Green arrows point to cell-surface protrusion association of F-actin, while the blue arrow shows an example of local actin concentration around a 2 µm diameter vesicle. Scale bars are 1 µm.

**Figure 4:**
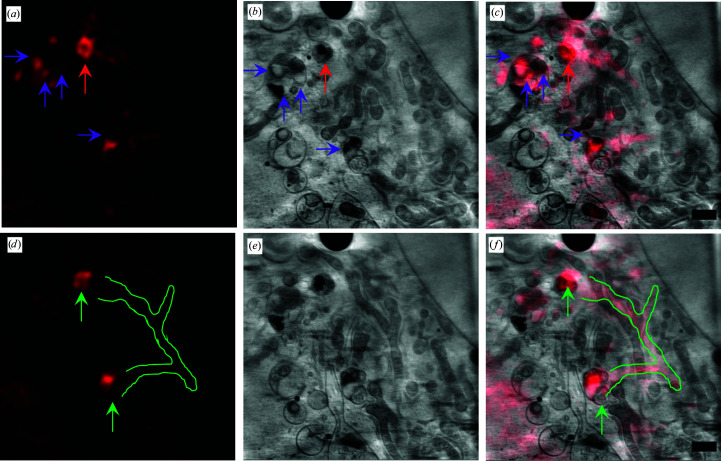
2D instances of SIM data (*a*, *d*) at different *z* heights, the corresponding SXT data (*b*, *e*) and the correlated data (*c*, *f*) for a perinuclear region. The SXT data shown here were collected using the 25 nm zone plate. Red arrows indicate F-actin-covered vesicles that appear connected through an F-actin network delineated with red boundaries. Purple arrows indicate the presence of F-actin coated subvesicles included in MVBs. Scale bars are 1 µm.

Bringing the two data sets together *in silico* allowed us to fully explore the prevalence of F-actin in areas of the cytoplasm that had clear organelle substructure both at the periphery of cells (Fig. 3 ▸) and in the near-perinuclear area (Fig. 4 ▸). The SXT data sets in Figs. 3 ▸ and 4 ▸ were collected using the 40 and 25 nm resolution zone plates, respectively, resulting in fields of view of 16^2^ and 10^2^ µm^2^, respectively. F-actin networks can be seen formed by the cell membrane, in filopodia-like cell extensions (Fig. 3 ▸) and even around and within individual cytoplasmic vesicles (Figs. 3 ▸ and 4 ▸). Bundles of F-actin filaments have also been found to traverse the cytoplasm just above concentrations of organelles and associated structures (Fig. 3 ▸). Nonetheless, without the correlated fluorescence signal to confirm the presence of actin, it would not have been possible to conclusively identify the observed structure by SXT. We have therefore been able to productively couple SXT and SIM and image F-actin *in cellulo* within cells in a near-native state. A segmentation of the visible actin bundles in the SXT data used in Fig. 4 ▸ has been attempted and is shown in Supplementary Fig. S1.

### 3.2. Impact on biological insights

This work was driven by the need to better understand the F-actin superstructure at key places within the cellular landscape. The method described here aimed to identify areas of biological interest and provide a methodological platform to further untangle the molecular basis of our observations. In this way, F-actin was found to be localized primarily at cell edges and in particular where a cell was extending to increase its contact with neighbours (Fig. 3 ▸). This was by no means unexpected given that F-actin has traditionally been associated with plasma-membrane plasticity and cell motility (Bisaria *et al.*, 2020 ▸; Buracco *et al.*, 2019 ▸; Schaks *et al.*, 2019 ▸). However, F-actin tropism becomes more diverse as we focus on the perinuclear area, where larger F-actin structures are found coincident with cellular vesicles such as endosomes and multivesicular bodies (MVBs) (Figs. 3 ▸ and 4 ▸). F-actin filaments can be observed completely surrounding a single relatively large vesicle (2 µm in diameter; Fig. 3 ▸) or contained within MVBs and associated with distinct substructures within them (Fig. 4 ▸), in accordance with previous reports implicating F-actin in membrane plasticity, vesicle formation (Picco *et al.*, 2018 ▸; Villanueva *et al.*, 2016 ▸) and MVB biogenesis (Traikov *et al.*, 2014 ▸). Our data appear to confirm the direct involvement of F-actin in these events and are likely to point to areas of active membrane and receptor recycling during the formation and shuttling of MVBs. It is noteworthy that the events that we have captured in our imaging in U2OS cells, which are defined by the high concentration of ordered F-actin around or within a vesicle or MVB, are low in occurrence, with only a handful of distinct peri-vesicular localizations per cell imaged. This argues that such a recruitment process is only undertaken where and when needed in a cell, rather than presenting constitutively around all vesicles that are present in the cytoplasm. What controls such selectivity will be the subject matter of further investigations. In our data, F-actin structures are apparent traversing the length of elongated mitochondria and they display coincident branching at organelle branching points. This confirms that the F-actin network is implicated in mitochondrial trafficking leading to the fusion and fission events necessary for the health of the mitochondrial network of a cell (Villanueva *et al.*, 2016 ▸; Hoffmann *et al.*, 2019 ▸). Moreover, F-actin filaments appear to link vesicles with high incidence of F-actin to other endosome or MVB-like vesicles as well as to mitochondria. This suggests that the presence of actin molecular scaffolds plays a role in vesicular trafficking and cross-talk (Anitei & Hoflack, 2012 ▸). Intracellular transport had traditionally been claimed to depend solely on microtubules (the largest filaments of the cytoskeleton) before an actin-dependent mechanism was revealed (Schuh, 2011 ▸). In the actin-based transport method vesicles are thought to recruit actin nucleation factors, leading to the assembly of actin networks connecting the vesicles to each other and to the plasma membrane. We have therefore been able to capture an instance of the intracellular world that corroborates the active involvement of F-actin in vesicular processing and communication.

## 4. Conclusions

The work presented here demonstrates the role that a novel correlative 3D cryo-imaging scheme combining SIM and SXT can play in revealing the structure and function of F-actin organisation within mammalian cells. This is achieved by unambiguously correlating the presence of F-actin within the intracellular space, following imaging of the fluorescently tagged F-actin beyond the diffraction limit of visible light, while reaping the unique advantages provided by SXT in high-resolution imaging in near-native, whole and unstained cells. This correlative approach was used to visualize actin filaments in U2OS cells without the need for contrast-enhancing treatments or sample processing beyond snap-freezing. The correlated data provided high-resolution high-fidelity imaging in 3D of F-actin substructures and their spatial relationship within the cytoplasm, and confirmed both the conventional localization of F-actin in cell extremities and also its potential role in inter-vesicle and intra-vesicle communication and trafficking. Seen through our correlative approach, occurrences such as highly structured F-actin networks that link areas in the periplasmic space and F-actin filaments surrounding whole vesicles (within these networks) corroborate the multifaceted aspects of cellular life that depend on F-actin distribution and remodelling in response to environmental or intracellular cues.

Correlated super-resolution fluorescence microscopy and soft X-ray absorption tomography at cryogenic temperatures as implemented at beamline B24 perfectly offer a complementary approach to mesoscale bio-imaging which delivers imaging data of low-contrasting cellular features that are otherwise hard to acquire. In this way it can unlock new potential in biological imaging and allow the holistic investigation of cellular structures.

## Supplementary Material

Supplementary Figure S1. DOI: 10.1107/S2059798321010329/qt5004sup1.pdf
