# Supplementary material for: Correlative cryo-imaging of the cellular universe with soft X-rays and laser light used to track F-actin structures in mammalian cells
Source: Acta Crystallogr D Struct Biol. 2021 Nov 29;77(Pt 12):1479–85. doi: 10.1107/S2059798321010329 (PMC8647181; doi:10.1107/S2059798321010329)
Supplement: Supplementary file 1 [file d-77-01479-sup1.pdf]

STRUCTURAL
BIOLOGY

Volume 77 (2021)

Supporting information for article:

Correlative cryo-imaging of the cellular universe with soft X-rays and laser light used to track F-actin structures in mammalian cells

Mohamed Koronfel, Ilias Kounatidis, Dennis M. Mwangangi, Nina Vyas, Chidinma Okolo, Archana Jadhav, Tom Fish, Phatcharin Chotchuang, Albert Schulte, Robert C. Robinson and Maria Harkiolaki

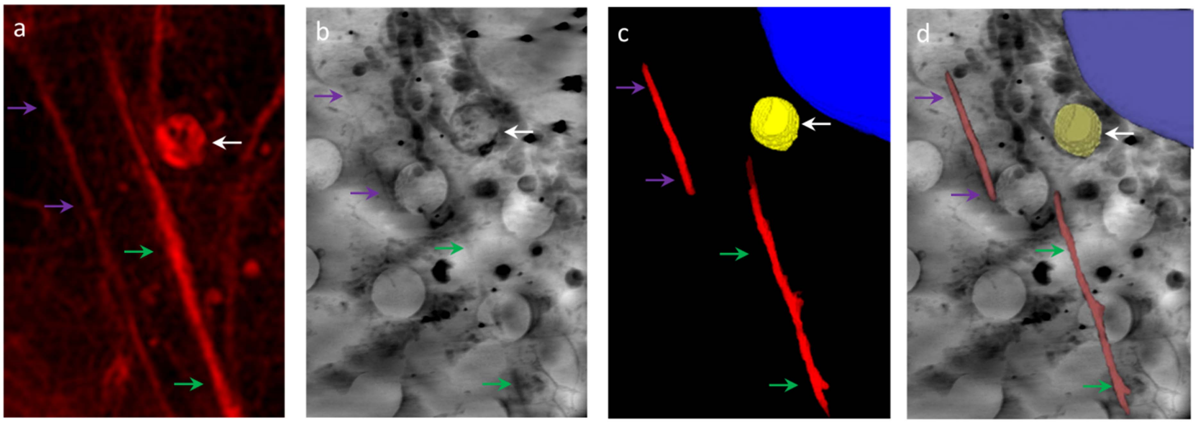

Figure S1
